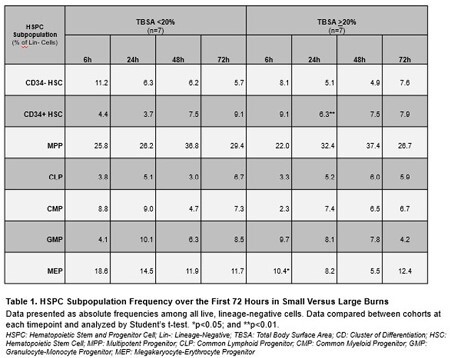# 20 Large Burn Injury Leads to Pathological Alteration of Hematopoietic Stem and Progenitor Cell Lineage Commitment

**DOI:** 10.1093/jbcr/irae036.020

**Published:** 2024-04-17

**Authors:** Ryan M Johnson, Kevin E Galicia, Huashan Wang, Mashkoor A Choudhry, John Kubasiak

**Affiliations:** Loyola University, Chicago, Illinois; Loyola University, Maywood, Illinois; Loyola University, Chicago, Illinois; Loyola University, Maywood, Illinois; Loyola University, Chicago, Illinois; Loyola University, Maywood, Illinois; Loyola University, Chicago, Illinois; Loyola University, Maywood, Illinois; Loyola University, Chicago, Illinois; Loyola University, Maywood, Illinois

## Abstract

**Introduction:**

Hematopoiesis follows a hierarchical differentiation process that begins with hematopoietic stem and progenitor cells (HSPC). This cascade culminates in the generation of erythroid, myeloid, and lymphoid lineages. Under pathological conditions, lineage commitment can be altered, and these changes are currently poorly understood in the setting of burn injury. This study seeks to provide a more comprehensive characterization of circulating HSPC in individuals who have sustained burn injuries with respect to burn size and the occurrence of sepsis.

**Methods:**

Flow Cytometry was utilized to analyze blood samples. Gating was performed with lineage markers CD3, CD14, CD19, CD41, CD56, and CD66c to determine all live, lineage-negative populations. Further gating was performed with markers CD34, CD38, CD45RA, CD49f, and CD135 to determine HSPC subpopulations. Patients were categorized into two groups based on the extent of their burn injuries: "Large" burns (>20% Total Body Surface Area or TBSA) and "Small" burns ( < 20% TBSA). They were also categorized into "Non-Septic" and "Septic" groups depending on whether they had negative (-) or positive (+) results in their blood cultures. To compare demographic, burn-related, and clinical data among these groups, statistical tests including the Student t-test, Fisher exact test, and Wilcoxon rank-sum test were employed as appropriate. HSPC event counts and frequencies were analyzed using standard flow cytometry software.

**Results:**

A total of 14 patients were included in this study, 50% of which sustained Large burns of TBSA >20%. The entire cohort had a mean age of 49.4 + 14.6 years, with a greater proportion of males (71.4%) and those identifying as non-white (71.4%). During the first 72 hours after injury, absolute HSPC frequencies were largely similar between Small and Large Burn cohorts (Table 1). Among the Large Burn cohort, relative frequency of common progenitors differed when comparing Non-Septic and Septic cohorts. Those developing sepsis experienced megakaryocyte and myeloid progenitor expansion, with a corresponding reduction in lymphoid progenitors (Figure 1).

**Conclusions:**

During the first 72 hours following injury, absolute HSPC frequencies appear to be largely similar when comparing small and large burns. However, when burn injury exceeds 20% TBSA, relative frequencies of common progenitors differ when comparing Non-Septic and Septic patients. Those who go on to develop sepsis display a predominance of megakaryocyte and myeloid progenitors; these patients also demonstrate a shift away from lymphopoiesis. These findings suggest that HSPC lineage commitment is pathologically altered in a subset of large burns, and the observed derangements correlate with the development of sepsis.

**Applicability of Research to Practice:**

Understanding the immunological derangements that occur in burn and sepsis can lead to targeted therapies that can drive the immune system in a more favorable direction.